# Molecular Characterization of *Salmonella enterica* Serovar Aberdeen Negative for H_2_S Production in China

**DOI:** 10.1371/journal.pone.0161352

**Published:** 2016-08-23

**Authors:** Fuli Wu, Xuebin Xu, Jing Xie, Shengjie Yi, Jian Wang, Xiaoxia Yang, Chaojie Yang, Beibei Liang, Qiuxia Ma, Hao Li, Hongbin Song, Shaofu Qiu

**Affiliations:** 1 Institute of Disease Control and Prevention, Academy of Military Medical Sciences, Beijing, China; 2 Shanghai Municipal Centre for Disease Control and Prevention, Shanghai, China; University of Minnesota, UNITED STATES

## Abstract

*Salmonella enterica* infections continue to be a significant burden on public health worldwide. The ability of *S*. *enterica* to produce hydrogen sulfide (H_2_S) is an important phenotypic characteristic used to screen and identify *Salmonella* with selective medium; however, H_2_S-negative *Salmonella* have recently emerged. In this study, the H_2_S phenotype of *Salmonella* isolates was confirmed, and the selected isolates were subjected to antimicrobial susceptibility testing and molecular identification by multilocus sequence typing, pulsed-field gel electrophoresis, and clustered regularly interspaced short palindromic repeat (CRISPR) analysis. The *phs* genetic operon was also analyzed. A total of 160 *S*. *enterica* serovar Aberdeen isolates were detected between 2005 and 2013 in China. Of them, seven non-H_2_S-producing isolates were detected. Notably, four samples yielded four pairs of isolates with different H_2_S phenotypes, simultaneously. The data demonstrated that H_2_S-negative isolates were genetically closely related to H_2_S-positive isolates. Three new spacers (Abe1, Abe2, and Abe3) were identified in CRISPR locus 1 in four pairs of isolates with different H_2_S phenotypes from the same samples. Sequence analysis revealed a new nonsense mutation at position 208 in the *phsA* gene of all non-H_2_S-producing isolates. Additionally, we describe a new screening procedure to avoid H_2_S-negative *Salmonella*, which would normally be overlooked during laboratory and hospital screening. The prevalence of this pathogen may be underestimated; therefore, it is important to focus on improving surveillance of this organism to control its spread.

## Introduction

*Salmonella enterica* is a common food-borne zoonotic pathogen found worldwide [1]. All non-typhoidal *Salmonella* (over 2,700 serovars) are considered human pathogens, with an estimated 93.8 million cases of infection, and up to 150,000 deaths a year annually in humans [2]. A substantial number of human diseases related to non-typhoidal *Salmonella* occur in developed countries [3, 4]. In China, an estimated 9.03 million cases of *S*. *enterica* infection are reported annually, and outbreaks are common [5]. In recent years, *S*. *enterica* serotype Aberdeen (*S*. Aberdeen) has been detected in Shanghai and Nanjing using our *Salmonella* surveillance system. This pathogen has a wide host range, infecting cattle and swine, however, only a few cases have been reported over the last 50 years [6–8]. *S*. Aberdeen virulence is inferior to that of other serotypes, which often cause sporadic disease [9]. *S*. Aberdeen mainly infects individuals with weakened immune systems, such as infants and young children, and causes abdominal pain and diarrhea as the primary clinical manifestations.

The ability to produce hydrogen sulfide (H_2_S) is an important phenotypic characteristic used for the screening and identification of *Salmonella* with selective medium, including deoxycholate hydrogen sulfide lactose (DHL), *Salmonella-Shigella* (SS), and triple sugar iron (TSI) agar [10]. However, a range of H_2_S-negative *Salmonella* serotypes, including *S*. Typhimurium, *S*. Infantis, *S*. Kentucky, *S*. Senftenberg, *S*. Enteritidis, *S*. Heidelberg, and *S*. Derby, have emerged recently in Japan, Kuwait, China, and Hong Kong [10–13]. Using our laboratory *Salmonella* surveillance system, 160 (7.3%) *S*. Aberdeen isolates were detected among 2,179 *Salmonella* isolates between 2005 and 2013 in China. Among these 160 isolates, seven (4.4%) were identified as non-H_2_S-producing *S*. Aberdeen. To the best of our knowledge, this is the first report of the identification of H_2_S-negative *S*. Aberdeen.

Therefore, in this report, we investigated genetic variations among non-H_2_S-producing and selected H_2_S-producing *S*. Aberdeen isolates by multilocus sequence typing (MLST), pulsed-field gel electrophoresis (PFGE), and clustered regularly interspaced short palindromic repeat (CRISPR) analysis [14–18], with the aim to clarify the molecular basis of the inability of these isolates to produce H_2_S.

## Materials and Methods

### Ethics statement

During our routine surveillance of *Salmonella*, fecal samples from individual outpatients with diarrhea were collected and screened in sentinel hospitals based on a national pathogen monitoring system. The study was approved and authorized by the institutional ethics committees of the Academy of Military Medical Sciences of the Chinese People’s Liberation Army (Beijing, China). The institutional review board of the Academy of Military Medical Sciences waived the requirement for written informed consent from the participants.

### Isolates

**Fig 1:**
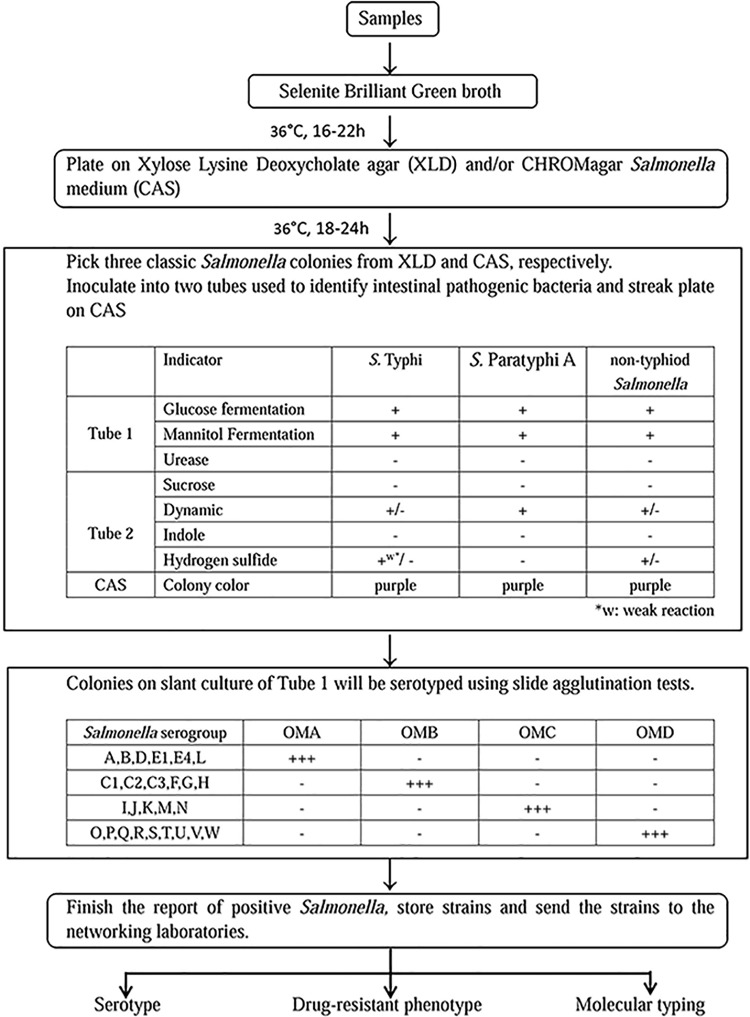
The methodology of *Salmonella* detection procedure.

A surveillance system of infectious diseases has been established in our laboratory. As the center monitoring laboratory in this system, we are responsible for collecting samples from network laboratories and sentinel hospitals. In our study, the vegetable, aquatic product, and swine manure were collected from markets of agricultural products from different districts in Shanghai, and water samples were collected from the Huangpu River. The samples were analyzed as described below. Our *Salmonella* detection procedure involved several processes: enrichment, isolation, species identification, and sero-typing. The overall methodology is presented as a flow diagram in Fig 1. Samples were added to Selenite Brilliant Green broth (CHROMagar, Shanghai, China) and incubated at 36°C for 16–22 h to enrich bacteria. The pre-enriched samples were plated on xylose lysine deoxycholate agar (XLD; CHROMagar) and CHROMagar *Salmonella* medium (CAS; CHROMagar) simultaneously, followed by incubation at 36°C for 18–24 h. Three classic *Salmonella* colonies were picked from XLD and CAS, inoculated into two tubes (Tube 1 and Tube 2; see Fig 1), used to identify intestinal pathogenic bacteria, and plated on CAS to confirm the H_2_S phenotype. The samples were then incubated at 36°C for 18–24 h. Colonies on Tube 1 slant cultures were serotyped using slide agglutination tests (SSI Diagnostica, Hillerød, Denmark). API 20E test strips (bioMérieux SA, Marcy l’Etoile, France) were used to confirm agglutinative and incomplete agglutinative bacterial colonies and to test for the H_2_S phenotype.

### Antimicrobial susceptibility testing

Antimicrobial susceptibility testing was carried out using automated broth microdilution (Sensititre; Thermo Fisher Scientific, USA). Twenty-one antibiotics, including amikacin, ampicillin, aztreonam, cefazolin, cefepime, cefoperazone, cefoxitin, ceftazidime, ceftriaxone, chloramphenicol, gentamicin, piperacillin, tetracycline, thienamycin, ticarcillin, ticarcillin/clavulanic acid, tobramycin, trimethoprim/sulfamethoxazole, levofloxacin, nitrofurantoin and norfloxacin, were used to test antimicrobial susceptibility. According to the breakpoints outlined by the Clinical and Laboratory Standards Institute (CLSI), each isolate was recorded as resistant or susceptible for each antimicrobial. The CLSI-specified susceptible control strain was *Escherichia coli* ATCC 25922.

### MLST analysis

MLST analysis was conducted according to the protocols described on the MLST website (http://mlst.ucc.ie/mlst/dbs/Senterica/documents/primersEnterica_html). Briefly, the *S*. Aberdeen isolates were subcultured into Luria broth (LB) at 37°C for 18 h; then, total DNA was extracted using a TIANamp bacteria DNA kit (Tiangen Biotech, Beijing, China) and stored at –20°C prior to use. Seven housekeeping genes (*thrA*, *purE*, *sucA*, *hisD*, *aroC*, *hemD*, and *dnaN*) were amplified by polymerase chain reaction (PCR) using primer sequences downloaded from the MLST database. PCR amplified products were sequenced by the BGI. The resulting sequence data were imported into the MLST database (http://mlst.warwick.ac.uk/mlst/mlst/dbs/Senterica) and information of the sequence type (ST) was obtained.

### PFGE analysis

PFGE was performed as previously described [19]. The purified total DNA was digested with *Xba*I (TaKaRa, Dalian, China) at 37°C for 3 h. DNA macrorestriction fragments were resolved over 20 h on 1% SeaKem gold agarose (Lonza, Rockland, ME, USA), in 0.5M Tris-borate-EDTA buffer, using a CHEF Mapper PFGE system (Bio-Rad, Hercules, CA, USA). The electrophoresis conditions had an initial switch time of 2.16 s, with final switch times of 63.8 s. *S*. *enterica* serotype Braenderup H9812 was used as the molecular size standard [20]. The gel images were digitally captured for analysis using BioNumerics software version 6.0 (Applied Maths, Belgium). The genetic similarity coefficients were calculated, and dendrograms were constructed by the unweighted pair group method of arithmetic average (UPGMA). The analysis parameters used in this study were based on 1.2% tolerance values.

### CRISPR analysis

Two CRISPR loci (CRISPR 1 and CRISPR 2) are reported to be present in all *Salmonella* genomes [21], and molecular epidemiological investigations have strongly correlated CRISPR polymorphisms with serotype [16]. CRISPR typing was performed between four paired isolates from the same samples with different H_2_S phenotypes. Primer pairs were selected based on a previous study [15]. PCR conditions were as follows: 95°C for 5 min; 30 cycles of 95°C for 30 s, 63°C for 40 s, and 72°C for 45 s; and 72°C for 7 min, using *Ex Taq* DNA polymerase (TaKaRa/Clontech, Beijing, China). PCR-amplified products were sequenced by the BGI. The resulting sequence data were imported into the CRISPRfinder Website (http://crispr.u-psud.fr/Server/) to obtain spacer and direct repeat information [21]. The name of each spacer was obtained using the Institute Pasteur CRISPR database for *Salmonella* (http://www.pasteur.fr/recherche/genopole/PF8/crispr/CRISPRDB.html) [13]. In cases where the spacer or direct repeat was unknown in the CRISPR dictionary, a new spacer name was assigned according to recognized spacer nomenclature [15].

### Sequence analysis of the *phs* operon

The *phs* operon is critical for the reduction of thiosulfate to hydrogen sulfide and contains three genes (*phsA*, *phsB*, and *phsC*) encoding thiosulfate reductases [22, 23]. To determine the molecular basis of the inability of some strains to produce hydrogen sulfide, the *phs* operon was amplified, and the resulting sequences were analyzed. PCR conditions were as follows: 95°C for 5 min; 30 cycles of 95°C for 30 s, 57°C for 40 s, and 72°C for 45 s; and 72°C for 7 min, using *Ex Taq* DNA polymerase (TaKaRa/Clontech). Primer pairs [13] are presented in Table 1. The sequence data obtained were imported into DNAman 6.0, and genetic differences were detected using Mega 6.0. The genome sequence of strain *Salmonella* Typhimurium LT2 (NC_003197.1) was used as a reference.

**Table 1:** Primers for PCR amplification of the *phsABC* genes.

*Gene*	*Primer*	*Products length*
*phsA1*	F 5'-CGTTGGATGCCTGTTCAG-3'	938
	R 5'-AGGTCGTAGAGCCGATTG-3'	
*phsA2*	F 5'-CGCCGTTCAACTGATAGA-3'	959
	R 5'-AATGGTGAGCTTCGATCC-3'	
*phsA3*	F 5'-CATCGTAGAGCTGTTCATCA-3'	975
	R 5'-CATGTGCGTGTTCAGGAA-3'	
*phsB*	F 5'-CAAGCATGAGCAGCACCAC-3'	687
	R 5'-ATGAGGGAGGAGGGAACCAT-3'	
*phsC*	F 5'-GATGGTCTCTATTTGCCGTTCT-3'	803
	R 5'-GGTGCTGCTCATGCTTGTT-3'	

### Nucleotide sequence accession numbers

The nucleotide sequences obtained in this study have been deposited in the NCBI under GenBank accession numbers KU143714–KU143732.

## Results

### Identification of *S*. Aberdeen isolates

In total, 160 *S*. Aberdeen isolates were detected in our laboratory from 2005 to 2013. Among these isolates, 58.75% were isolated from human cases, 30.63% were isolated from aquaculture products, 3.12% were isolated from vegetable, 3.12% were isolated from surface water, 2.51% were isolated from poultry, and 1.87% were isolated from swine manure. Additionally, 39 (24.37%) of 49 aquaculture products isolates were detected from *Ballamya quadrata* (spiral shell). We identified seven (4.4%) non-H_2_S-producing *S*. Aberdeen isolates. Notably, two stool samples yielded two pairs of isolates with different H_2_S phenotypes simultaneously (SH06084+ and SH06084–, SH11G1146+ and SH11G1146–; where + indicates an H_2_S-producing isolate and–indicates a non-H_2_S-producing isolate). Similar findings were obtained from one surface water sample (SH11SF112+ and SH11SF112–) and one vegetable sample (SH06SF12+ and SH06SF12–). According to molecular typing results, isolation origin, source, H_2_S phenotype, and isolation year, a total of 45 isolates, including 39 isolates from Shanghai and six isolates from Nanjing, were selected for further analysis, with seven non-H_2_S-producing isolates and 38 H_2_S-producing isolates (used as reference strains).

### Antimicrobial susceptibility testing

All 45 *S*. Aberdeen isolates tested were susceptible to all 21 antibiotics.

### MLST analysis

All 45 *S*. Aberdeen isolates belonged to the same sequence type, ST426. Previously, two *S*. Aberdeen isolates belonging to ST426 were reported in the MLST database; one was isolated from the UK (1934) and the other was isolated from Australia (2001). A strain in the MLST database isolated from China in 2008 (serotype unknown), belonging to ST653, was also identified as a single-locus variant of ST426.

### PFGE analysis

**Fig 2:**
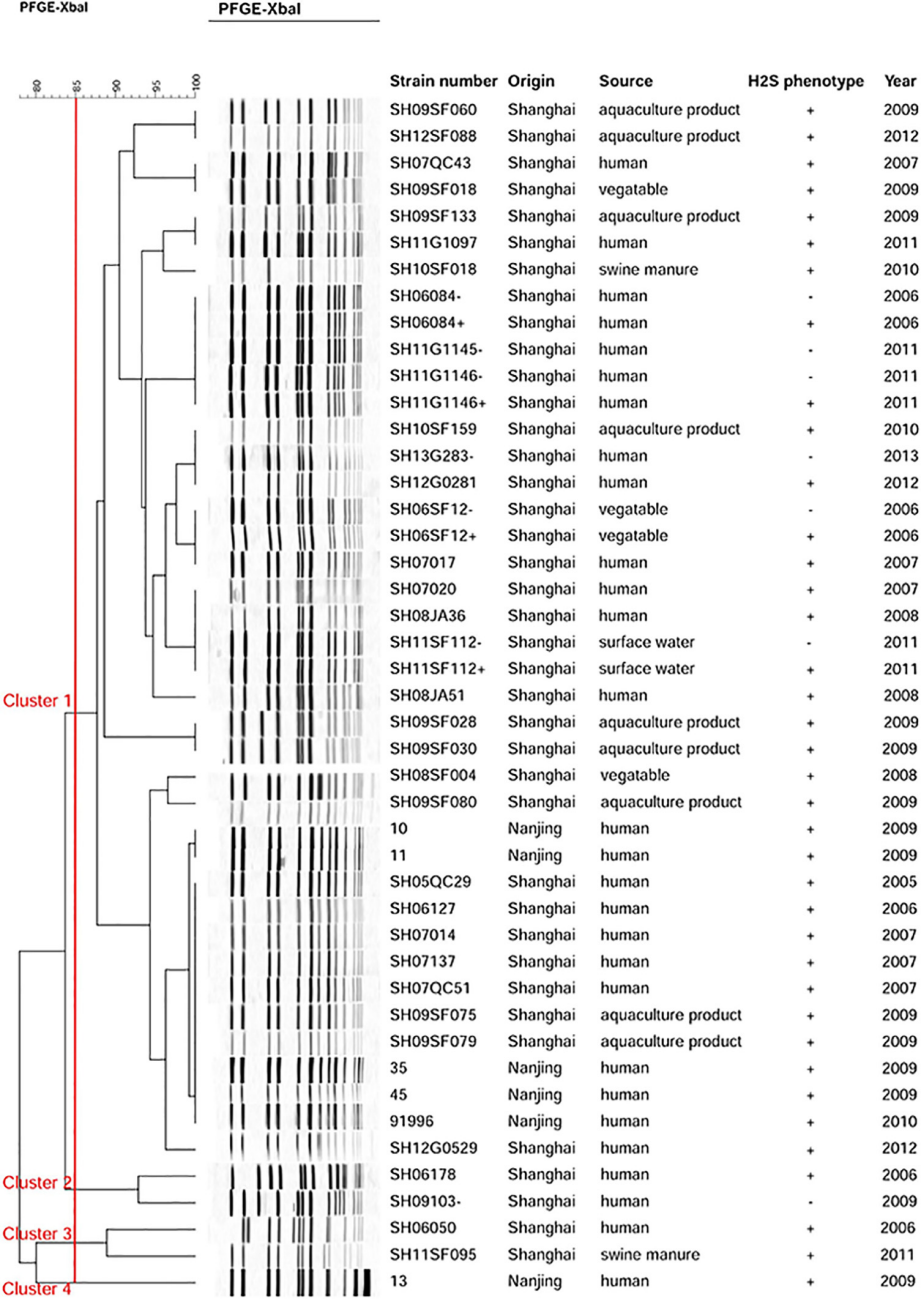
Dendrogram displaying the PFGE profiles of the 43 isolates. The strain number, origin, source, sequence type (ST), and H_2_S phenotype are shown for each strain. +, H_2_S-producing isolate; −, non-H_2_S-producing isolate.

PFGE analysis showed that 45 *S*. Aberdeen isolates had close genetic relationship, with 88.89% of the isolates belonging to a single clone and few isolates showing lesser relatedness. Cluster analysis primarily divided the isolates into four main clusters with approximately 85% similarity (Fig 2). Cluster 1, the largest cluster, contained 35 Shanghai isolates (including six H_2_S-negative isolates) and five Nanjing isolates. Most isolates from human cases gave the same PFGE banding patterns as isolates from the environment. SH10SF018, isolated from swine manure, was 96% similar to SH09SF133 and SH11G1097, isolated from aquaculture products and human cases, respectively. Two pairs of *S*. Aberdeen isolates from the same sample (SH11G1146+/–, SH06084+/–), and one H_2_S-negative isolate (SH11G1145–) gave the same PFGE banding pattern. Notably, one human isolate (SH07017) shared the same PFGE banding pattern as an H_2_S-negative isolate (SH06SF12–) from vegetables. Another non-H_2_S-producing isolate (SH13G283–) from 2013 displayed an identical PFGE profile to that of an H_2_S-producing isolate (SH12G0281) from 2012. In addition, SH11G112+ and SH11G112–, isolated from the same surface water sample, had the same PFGE profile as H_2_S-positive *S*. Aberdeen isolates from humans. Furthermore, the isolate SH09103–, identified in Cluster 2, was 92.86% similar to the human isolate SH06178. One *S*. Aberdeen isolate from a human in Cluster 3 was 88.89% similar to the other swine manure isolate, and Cluster 4 contained one isolate from Nanjing.

### CRISPR analysis

Four pairs of *S*. Aberdeen isolates from the same samples had the same spacer content in CRISPR 1 locus (STM1-Der3-Mik1-Abe1-Abe2-Abe3) and CRISPR 2 locus (ParBB1-AbeB1-AbeB2). There are currently no CRISPR 1 spacers designated for *S*. Aberdeen in the Institute Pasteur CRISPR database dictionary of spacers. In the four pairs of isolates, we identified three new spacers in the CRISPR 1 locus, namely spacers Abe1, Abe2, and Abe3. These four pairs of isolates shared the same CRISPR loci, indicating that they were genetically closely related.

### Sequence analysis of the *phs* operon

**Fig 3:**
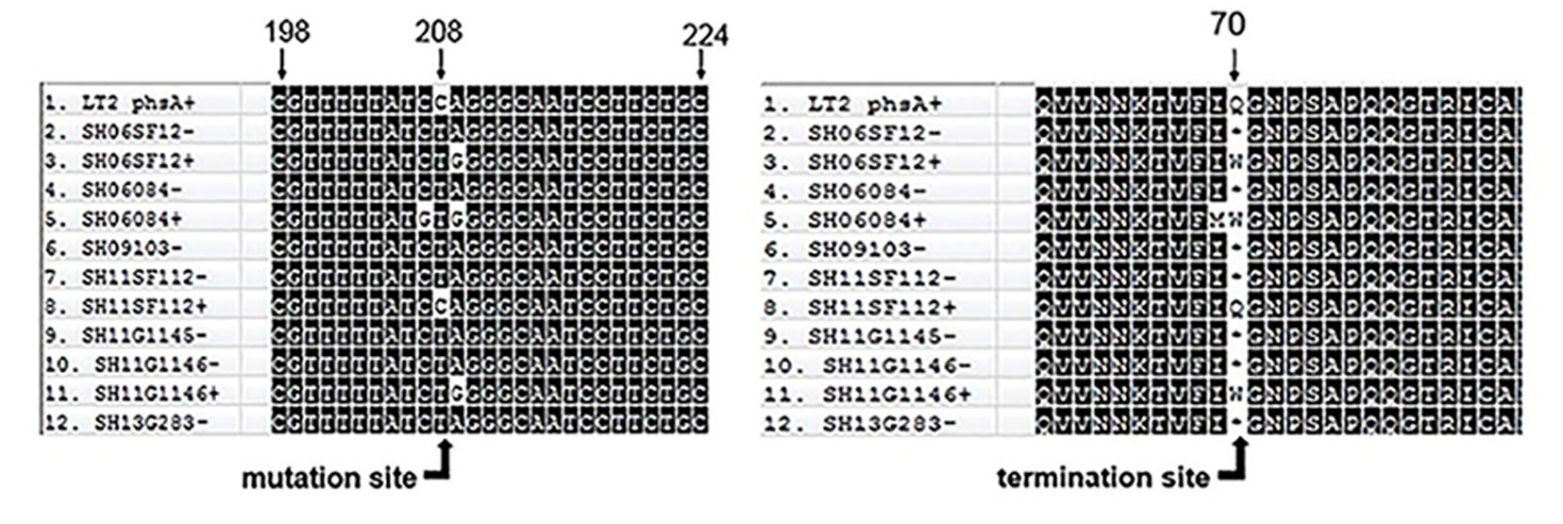
Sequence alignment of the *phs* gene and the protein. A nonsense mutation at position 208 of the *phsA* gene results in the replacement of a sense codon (CAG) with a termination codon (UAG) leading to the premature termination of *phsA*. The first sequence, *phsA*, is based on *S*. enterica serotype Typhimurium strain LT2 (GenBank AE006468). *, termination codon; +, H_2_S-producing isolate; −, non-H_2_S-producing isolate.

We identified a new mutation site present in seven non-H_2_S-producing *S*. Aberdeen isolates. Compared with 38 H_2_S-producing *S*. Aberdeen isolates, the seven non-H_2_S-producing *S*. Aberdeen isolates displayed a single-nucleotide substitution of C to T at position 208 of the *phsA* gene, resulting in a codon change from CAG to UAG. The replacement of a sense codon (CAG) with a termination codon (UAG) led to the premature termination of *phsA* (Fig 3), resulting in the inability of non-H_2_S-producing *S*. Aberdeen isolates to produce integral *phsA* gene products. Although isolates SH06SF12+, SH06084+, and SH11SF1146+ had a mutation at position 208 of *phsA*, causing replacement of glutamine with tryptophan, this did not lead to inactivation of *phsA* gene products and thus did not alter the H_2_S phenotype. Mutations in *phsB* and *phsC* were not detected in the seven non-H_2_S-producing isolates (data not shown).

## Discussion

Based on our laboratory surveillance system, a total of 160 *S*. Aberdeen isolates were detected from Shanghai and Nanjing. Over half of these strains (58.75%) were isolated from human cases, whereas 24.37% were isolated from spiral shell. Our monitoring data, collected between 2006 and 2011 [7], demonstrated that *S*. Aberdeen could be enriched by spiral shells. Thus, it is likely that the spiral shell is a potential host of *S*. Aberdeen. Moreover, spiral shells have become a popular food in Shanghai in recent decades. Typically, spiral shells are stored in wet, aerobic conditions, and spiral shell emunctory organs are often cut before the snails are cooked; both of these actions promote *S*. Aberdeen growth. A previous study suggested that animal contact is a source of human non-typhoidal salmonellosis and that *Salmonella* could be parasitic in livestock intestines [24]. Furthermore, two isolates were detected from swine manure, which is often directly excreted into surface water, causing contamination over a period of several years. Thus, we speculate that the spread of *S*. Aberdeen in China may be related to the inappropriate management of aquaculture and surface water. Additionally, clinical cases are most likely to occur from contaminated food. Therefore, the chain of *S*. Aberdeen transmission may proceed as follows: swine manure→surface water→spiral shells→humans suggesting that improved monitoring of aquaculture products and surface water is necessary in order to avoid contamination with *S*. Aberdeen, which may lead to infection in humans and animals.

Seven non-H_2_S-producing isolates were recovered in this study; five of these isolates were from clinical samples, one was from surface water, and one was from a vegetable. To the best of our knowledge, this is the first report of the identification of H_2_S-negative *S*. Aberdeen. Moreover, we identified four pairs of *S*. Aberdeen isolates with different H_2_S phenotypes from the same samples. These four non-H_2_S-producing *S*. Aberdeen isolates had sequence types, PFGE banding patterns, and CRISPR spacers identical to those of four H_2_S-producing isolates from the same samples, all indicating a close genetic relationship. Taken together, the molecular typing analysis and antibiotic susceptibility testing suggested that all the *S*. Aberdeen isolates identified in our laboratory from 2005 to 2013 had the same antibiotic susceptibility, belonged to the same ST, and had closely related PFGE patterns, indicating that these *S*. Aberdeen isolates were highly clonal.

H_2_S-negative *S*. Typhimurium, *S*. Infantis, and *S*. Senftenberg isolates have been reported to contain nonsense mutations at positions 1,440, 358, and 1,621 of the *phsA* gene, respectively [10, 13]. A nonsense mutation in the *phsA* gene was also identified in low-H_2_S-producing *S*. Typhi and *S*. Paratyphi A in a previous study [25]. Moreover, analysis of H_2_S-positive versus an H_2_S-negative variant *S*. Kentucky, isolated by researchers in Kuwait [11], showed a *moaC* insertion frameshift mutation, affecting molybdenum cofactor biosynthesis protein C. In our study, a new mutation site within the *phsA* gene was detected. The *phsA* gene, one of three open reading frames of the *phs* operon, is homologous and very similar to several other anaerobic molybdoprotein oxidoreductases. Seven non-H_2_S-producing *S*. Aberdeen isolates possess the same mutation at position 208 of *phsA*, resulting in a sense codon (CAG) being replaced by a termination codon (UAG; Fig 3). Therefore, the non-H_2_S-producing *S*. Aberdeen isolates cannot produce the integral *phsA* gene product, i.e., thiosulfate reductase, and are thus unable to utilize thiosulfate (S_2_O_3_^2−^) to produce H_2_S. Owing to the lack of an H_2_S phenotype, H_2_S-negative *Salmonella* are more likely to be overlooked during laboratory and hospital screening. Accordingly, because the detection methods used for H_2_S-negative *Salmonella* isolates are important for preventing the future spread of these pathogens, we have proposed a standard screening procedure (Fig 1) involving enrichment, isolation, species identification, and serotyping to avoid missing or misidentifying H_2_S-negative *Salmonella* isolates.

*Salmonella* triggers inflammation in the gut by using its virulence factors T3SS-1 and T3SS-2; at the same time, the host stimulates oxidation of endogenous sulfur compounds and converts thiosulfate (S_2_O_3_^2−^) to tetrathionate (S_4_O_6_^2−^) [26]. Previous studies have shown that anaerobic respiration based on tetrathionate, generated by oxidizing thiosulfate, provides a growth advantage for *Salmonella* to compete with other microbiota in the inflamed intestine [27–29]. Moreover, *Salomonella* can only use ethanolamine as a carbon source to support growth under anaerobic conditions in the presence of tetrathionate as a respiratory electron acceptor [30]. Thus, H_2_S-negative *Salmonella*, which are unable to convert thiosulfate to H_2_S, may cause increased in tetrathionate-dependent respiration, promoting *Salmonella* growth in the gut [10, 31]. More comprehensive studies are needed to analyze the virulence and pathogenicity of H_2_S-negative *Salmonella*.

Multiple serotypes of H_2_S-negative *Salmonella* had been detected in Japan, Kuwait, China, and Hong Kong [10–13]. Seventeen H_2_S-negative *S*. Senftenberg isolates [13] and 19 H_2_S-negative *S*. Choleraesuis isolates were reported in our previously study [32]. Here, we identified seven H_2_S-negative *S*. Aberdeen isolates in China from 2006 to 2011. Therefore, it is possible that H_2_S-negative *S*. *enterica* has been overlooked during hospital and laboratory screening, necessitating improved monitoring of this pathogen to prevent and control its spread.
